# Efficacy and Safety of a High-Energy, Low-Protein Formula Replacement Meal for Pre-Dialysis Chronic Kidney Disease Patients: A Randomized Controlled Trial

**DOI:** 10.3390/nu15214506

**Published:** 2023-10-24

**Authors:** Wen-Ching Yang, Hui-Min Hsieh, Jun-Peng Chen, Shang-Feng Tsai, Hsien-Fu Chiu, Mu-Chi Chung, Shih-Ting Huang, Yun-Yu Chen, Cheng-Hsu Chen

**Affiliations:** 1 Department of Food and Nutrition, Taichung Veterans General Hospital, Taichung 407219, Taiwan; 940403@gmail.com (W.-C.Y.); hmhsieh@vghtc.gov.tw (H.-M.H.); 2 Biostatistics Group, Department of Medical Research, Taichung Veterans General Hospital, Taichung 407219, Taiwan; pippan7676@vghtc.gov.tw (J.-P.C.); r01847021@gmail.com (Y.-Y.C.); 3 Division of Nephrology, Department of Internal Medicine, Taichung Veterans General Hospital, Taichung 407219, Taiwan; s881056@gmail.com (S.-F.T.); hsienfuchiu@gmail.com (H.-F.C.); mcchung@vghtc.gov.tw (M.-C.C.); kitheroborn@hotmail.com (S.-T.H.); 4 Department of Life Science, Tunghai University, Taichung 407224, Taiwan; 5 Department of Medicine, National Yang-Ming Chiao Tung University, Taipei 112304, Taiwan; 6 Department of Post-Baccalaureate Medicine, College of Medicine, National Chung Hsing University School of Medicine, Taichung 40227, Taiwan

**Keywords:** oral nutritional supplement, low-protein diet, chronic kidney disease, meal replacement

## Abstract

High-energy, low-protein formulas (HE-LPFs) are commonly used as oral nutritional supplements (ONSs) to help provide extra calories to patients who are adhering to a low-protein diet (LPD) after diagnosis with chronic kidney disease (CKD). This randomized controlled trial aimed to evaluate the efficacy and safety of an HE-LPF as either a partial or a total replacement for one meal in pre-dialysis CKD patients. Stage 4–5 CKD patients received either a once-daily HE-LPF (HE-LPF group) or normal food (control group) for a period of 4 weeks while following an LPD. Overall, 73 patients who completed the study were included in the intention-to-treat population. After analyzing the 3-day food records, the HE-LPF group experienced a significant decrease in the percentage of energy derived from protein (*p* < 0.05) and an increase in the percentage of energy derived from fat (*p* < 0.05) compared to the control group. The two groups had no significant differences in body weight, body composition, grip strength, renal function, electrolytes, or metabolic markers. The HE-LPF group had a high adherence (94.9% at week 4), and no adverse effects were observed. HE-LPFs are safe to employ as meal replacements for pre-dialysis CKD patients adhering to an LPD.

## 1. Introduction

Chronic kidney disease (CKD) is a common chronic condition with an estimated global prevalence of approximately 10% [1]. In recent years, the medical strategy for CKD has included recommendations for cures for comorbidities, the prevention of complications during treatment, the avoidance of nephrotoxic drugs, and proper dietary management [2,3]. Dietary modification was previously shown to reduce the accumulation of uremic toxins and excess minerals, such as potassium and phosphates. These benefits not only improve symptomatic relief but also slow the loss of renal function, thus delaying the onset of dialysis [4].

One of the foundations of this therapeutic approach is the restriction of protein intake to levels of approximately 0.55–0.6 g/kg/day in nondiabetic CKD patients and 0.6–0.8 g/kg/day in diabetic CKD patients [5,6]. The success of implementing nutritional interventions in CKD patients is dependent upon the patient’s adherence to a low-protein diet (LPD). Protein restriction changes the eating habits of CKD patients and must, therefore, be intensively followed by a renal dietitian [7]. LPD implementation is both patient-centered and personalized to help with compliance, efficacy, and safety, and it involves referral from a nephrologist to a dietitian as medically prescribed care [8]. Furthermore, using a simplified and easy-to-follow dietary regimen may improve one’s adherence to an LPD [9]. The energy requirements in CKD are recommended to be 25–35 kcal/kg/day [5]. Poor appetite induced by uremia and protein restriction may lead to insufficient caloric intake, with these factors possibly causing protein–energy wasting (PEW) [10]. In addition to LPD control, individual diet prescriptions can be implemented in pre-dialysis CKD patients for one or more of sodium, phosphorus, and potassium, as determined by electrolyte or renal function [11]. There is no adequate energy supply in the prescription of a CKD diet; hence, oral nutritional supplements (ONSs) are considered a next step [12].

Previous research has shown that low-protein ONSs can improve both adherence to an LPD and nutritional status during pre-dialysis CKD [13,14,15,16]. Currently, the use of ONSs as complete or partial meal replacements has been studied, including diabetes-specific ONSs for improving glycemic control in diabetes patients [17] and high-protein ONSs for weight control in obese patients [18,19]. However, few studies have investigated low-protein ONSs in pre-dialysis CKD patients. The aim of this study was to evaluate the efficacy and safety of a high-energy, low-protein formula (HE-LPF) that was tailored to suit the nutritional management approach for pre-dialysis CKD patients as either a complete or a partial meal replacement in terms of changes in anthropometric measurements, dietary intake, clinical parameters, adherence, and adverse events.

## 2. Materials and Methods

### 2.1. Study Design

This study was a single-center, prospective, randomized, open-label, controlled, parallel-group trial that was carried out with pre-dialysis stage 4–5 CKD patients during a 4-week study period. Patients were recruited over a period of 8 months from the end-stage renal disease program database within the renal clinical service department of Taichung Veterans General Hospital during the period from April to December 2020. Eligible participants were assigned at a 1:1 ratio to either the HE-LPF group or the control group using computer-generated randomization to receive either the study intervention or a control comparator. The study was carried out according to the current version of the Declaration of Helsinki and Good Clinical Practice (GCP) guidelines, with reference to a study protocol approved by the Institutional Review Board of Taichung Veterans General Hospital (IRB approved number SF20045B). Signed and dated informed consent was obtained from all patients prior to enrollment in the study. The trial was registered at ClinicalTrials.gov (NCT05330663).

### 2.2. Subjects

Subjects meeting the following inclusion criteria were eligible for study: aged between 20 and 80 years, having an eGFR of <30 mL/min/1.73 m^2^ at screening, and having followed a low-protein diet that provided a protein level of 0.6–0.8 g/kg/day for at least 3 months prior to inclusion. The exclusion criteria were incident or prevalent dialysis, potential for renal transplantation, a body mass index (BMI) of <18 or >30 kg/m^2^, pregnancy or lactation, malnutrition with albumin levels of <3 g/dL with the need for caloric and other nutritional supplements, severe liver disease, malignancy or infectious disease, existing gastrointestinal disease or pathological findings contraindicating enteral nutrition (including intestinal ileus, acute upper gastrointestinal bleeding, malabsorption or maldigestion conditions, or previous significant surgical resection of the intestine, as well as dysphagia or high risk of aspiration), severely impaired gastrointestinal function (including severe constipation or acute diarrhea), relevant central nervous system and/or psychiatric disorders, known allergies or intolerance to any ingredient in the study product, planned surgery or hospitalization during the study period, suspicion of drug abuse, participation in another clinical trial within 30 days of the study’s start and for the duration of the study, and the inability to comply with the study’s instructions and procedures.

### 2.3. Dietary Management

A registered dietitian calculated the individual nutritional requirements for each patient in order for them to obtain the required 0.6–0.8 g/kg/day of protein and 25–35 kcal/kg/day of energy using their actual body weight according to clinical practice guidelines for the dietary management of CKD [5]. Keto-analogues were used where clinically appropriate on an individual patient basis to ensure an adequate supply of nitrogen-free precursors of essential amino acids. Before participating in the study, subjects were asked to maintain their usual CKD management behaviors and lifestyle (e.g., medications and exercise). Subjects received diet counseling from the same registered dietitian at the beginning of the study and again at 2 weeks to maximize the achievement of their nutritional goals.

### 2.4. Study Intervention

The intervention group was given a replacement for a single, typically high-protein meal or part of a meal (preferably breakfast) according to their dietary habits during the day. The HE-LPF consisted of 200 mL units (Fresubin^®^ Renal Drink providing 400 kcal, 6% of energy from protein, and reduced electrolytes, and containing fish oil and dietary fiber) with the composition shown in Supplementary Table S1. The control group received normal food (conventional care) during the 4-week study period. All patients returned to follow-up at week 2 and week 4 with 3-day food records. The dietitian then recommended a diet prescription according to the dietary habits of each patient. All patients in both groups continued to follow an LPD.

### 2.5. Efficacy Assessments

The efficacy endpoint was analyzed according to anthropometric indicators and dietary intake. The primary endpoint of efficacy was a change in body weight. Anthropometric measurements of body composition, grip strength, and waist circumference were all used to assess nutritional status. Body composition was determined using the Tanita MC-780 (TANITA Corporation, Akita, Japan) bioelectric impedance analysis technology, which involved measurement of body weight (BW, kg), body mass index (BMI, kg/m^2^), percentage body fat (%), fat mass (kg), muscle mass (kg), extracellular water (kg), and fat-free mass (kg). Grip strength was measured using a TTM-YD model hand dynamometer (Tsutsumi industries, Tokyo, Japan). Waist circumference was measured at the level of the iliac crest while the subject breathed minimally. The intake of energy, protein, carbohydrates (CHOs), saturated fatty acids (SFAs), monounsaturated fatty acids (MUFAs), polyunsaturated fatty acids (PUFAs) (eicosapentaenoic acid (EPA), and docosahexaenoic acid (DHA)), and the main minerals (sodium, potassium, calcium, phosphorus, and magnesium) was evaluated using 3-day food records. Energy and nutrients were calculated using the e-Kitchen system (2017 version, with the Taiwan Food and Drug Administration reference tables for food composition).

### 2.6. Safety and Adherence Assessments

The safety endpoint was analyzed using biochemical parameters, adherence, and adverse events. Biochemical parameters were collected using blood, including blood nitrogen urea (BUN), creatinine (Cr), sodium, chloride, potassium, calcium, phosphorus, magnesium, albumin, prealbumin, total protein, C-reactive protein (CRP), cholesterol, triglycerides, uric acid, low-density lipoprotein (LDL), and glycated hemoglobin (HbA1C). The eGFR was calculated using the Chronic Kidney Disease Epidemiology Collaboration (CKD-EPI) formula [20]. A 24 h urine sample was collected to measure urine protein and urine urea nitrogen (UUN), and the Maroni equation was used to calculate dietary protein intake [21]. Adverse events were evaluated according to gastrointestinal symptoms (including diarrhea, abdominal distention, constipation, nausea, and vomiting). Adherence to the prescribed interventional product was evaluated at weeks 2 (the first two weeks) and 4 (the final two weeks). To calculate adherence, patients in the intervention group kept a diary of their actual consumption of the food eaten within their low-protein diet. According to an evaluation by their dietitian and the nutritional content of the chosen meal, an interventional drink was included as either a complete or partial meal replacement.

### 2.7. Statistical Analysis

The calculated sample size involved 78 subjects based on a power of 80% using ANOVA: repeated measures between factors, two groups, three time points, and an effect size of 0.25 at the α = 0.05 significance level. The sample size was estimated using G * Power version 3.1.9.7 (a program written by Franz Faul, Universität Kiel, Germany) [22]. Assuming an approximately 5% dropout rate, we estimate that 84 patients were enrolled in the study. Data are presented as means (standard deviation; SD) and counts (percentages; %). The intention-to-treat (ITT) population was defined as subjects who completed all visits. The per-protocol (PP) population was defined as patients in the HE-LPF group who planned to use one bottle a day of an HE-LPF ONS to replace a high-protein meal or part of a meal, with adherence exceeding 85%. Analysis of the efficacy and safety outcomes in the ITT and PP populations was performed. Exploratory statistics included the Mann–Whitney U test for quantitative data and the chi-squared/Fisher exact tests for qualitative data. The primary outcome of the change in body weight was analyzed using a covariance model (ANCOVA) with the baseline body weight as the covariate and group assignment as the fixed class effect. The changes in body weight from baseline to week 2 and from baseline to week 4 were analyzed while controlling for confounders (estimated mean change expressed as the 90% confidence interval). The efficiency and safety of the outcome were analyzed with a mixed model for repeated measurements. The mixed model included the baseline value (day 1) of the results as the covariate and the two groups (HE-LPF and control) and the visit times (week 2 and week 4) as the fixed class effects. The covariance pattern was assumed to be unstructured but the same for both groups. A *p*-value of <0.05 was considered statistically significant. All data analyses were performed using IBM^®^ SPSS^®^ version 22.0 (International Business Machines Corp., New York, NY, USA).

## 3. Results

### 3.1. Participant Characteristics

**Figure 1:**
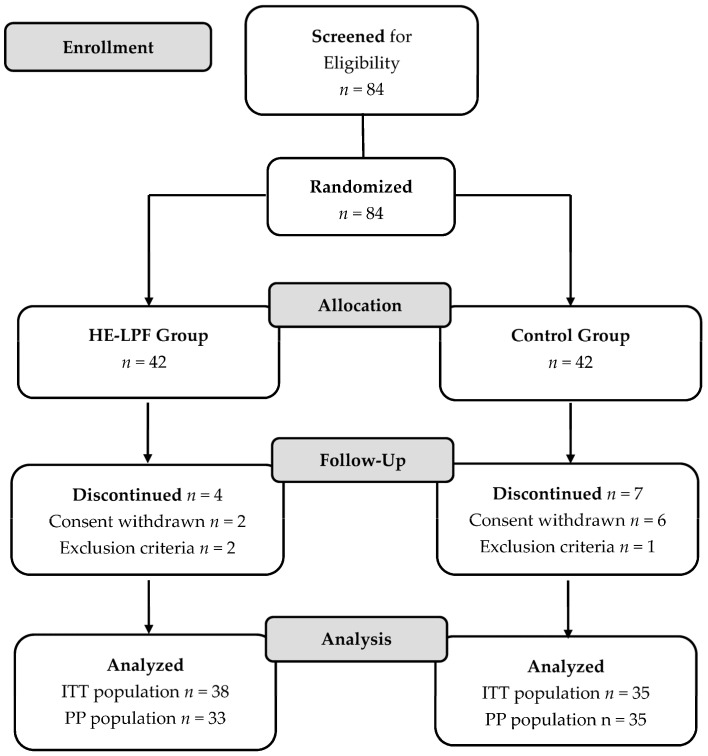
CONSORT flow diagram for trial patients, distinguishing between ITT and PP populations. In the ITT population, 11 patients were discontinued: in the HE-LPF Group, consent withdrawn, *n* = 2; exclusion criteria, *n* = 2, one severe liver disease and one albumin < 3. In the Control Group, consent withdrawn, *n* = 6; exclusion criteria, *n* = 1, one albumin <3. In the PP population, five patients were discontinued: <85% adherence with the HE-LPF, *n* = 5 in the HE-LPF Group.

The flowchart in Figure 1 describes the recruitment of participants from the ITT and PP populations. Of the 84 patients, all were eligible for inclusion in the study and subsequently randomized to either the HE-LPF group (*n* = 42) or the control group (*n* = 42). During the study, 11 patients were discontinued from the trial in the ITT population. In the HE-LPF group, two patients withdrew their informed consent, while two others were discontinued based on the exclusion criteria. One of these patients had severe liver disease, and the other had an albumin level below 3. In the control group, six patients discontinued their participation due to withdrawal of informed consent, while one was discontinued due to exclusion criteria, specifically an albumin level below 3. In the PP population, five patients were excluded after four weeks due to insufficient compliance (<85%) with the HE-LPF, resulting in a final inclusion of 33 participants from the HE-LPF group.

Table 1 shows the baseline characteristics of the ITT population. There was a small increase in eGFR in eight patients (three in the HE-LPF group and five in the control group in the range of 30–35 mL/min/1.73 m^2^) resulting from the slight variability in eGFR that is inherent in the repeated clinical monitoring of renal function. The subgroup analysis revealed that the incorporation of these patients with this deviation from the inclusion criteria did not lead to a statistically significant difference in the results. Patients were also matched for any concomitant medications being taken for the management of their symptoms related to CKD, with 9 patients in each group taking angiotensin-converting enzyme inhibitors or angiotensin receptor blockers (23.7% in the HE-LPF group and 25.3% in the control group) and 12 patients in each group taking keto-analogues (Ketosteril^®^, Fresenius Kabi GmbH) (HE-LPF group 31.6%, control group 34.3%). The HE-LPF and control groups matched in terms of all parameters.

### 3.2. Efficacy Outcomes

Table 2 shows the analysis of the efficacy outcomes in terms of body composition, waist circumference, and grip strength from baseline to 2 weeks and 4 weeks. The primary endpoints in body weight remained stable in both groups from baseline to 2 weeks, as well as at 4 weeks, with an equal change seen in the HE-LPF and control groups between baseline and week 4 (mean difference = −0.15 kg, 90% CI: −0.79 to 0.48, *p* = 0.685). ANCOVA did not show significant differences in body weight change between the two groups. Similarly, there were no differences in body weight, BMI, waist circumference, body composition, or grip strength during the 4 week study period. The PP population had the same results, and no variables differed between the two groups (Supplementary Table S2).

Table 3 shows an analysis of the efficacy outcomes regarding estimated energy, macronutrients, fatty acids, and major minerals based on the 3-day food records day food records from baseline to 2 weeks and 4 weeks. The energy intake (kcal/kg BW/day) in the ITT population was not significant, while in the PP population, it increased in the HE-LPF group (*p* = 0.028). Concerning macronutrient intake, the HE-LPF group exhibited a significant decrease in the percentage of energy derived from protein for the ITT (*p* = 0.021) and PP populations (*p* = 0.007). Additionally, there was a notable increase in the percentage of energy obtained from fat for the ITT population (*p* = 0.010) and the PP population (*p* = 0.013) when compared to the control group. In terms of fatty acid intake, the ITT population increased their intake of SFAs, MUFAs, and EPA (*p* = 0.032, *p* < 0.001, and *p* = 0.005, respectively), while the PP population increased their intake of SFAs, MUFAs, PUFAs, and EPA (*p* = 0.008, *p* < 0.001, *p* = 0.045, and *p* = 0.008, respectively). In terms of major mineral intake, the ITT and PP populations did not differ between the two groups.

### 3.3. Safety and Adherence Outcomes

**Figure 2:**
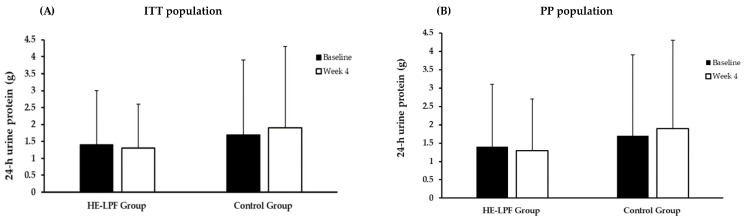
Comparison of 24 h urine protein levels at baseline and after four weeks in the HE-LPF Group and Control Group, (**A**) ITT population and (**B**) PP population.

The safety outcomes in terms of the biochemical indicators of renal function, serum electrolytes, plasma protein, metabolic markers, and 24 h urine collection from baseline to 2 weeks and 4 weeks are shown in Table 4 and Table 5. The data indicated no significant differences between the two groups in the ITT population (Table 4). A similar outcome was found in the PP population (Table 5). Although there were no statistically significant differences in 24 h urine protein levels between or within groups, the HE-LPF group showed a slight decrease following the four-week intervention. In contrast, the control group observed a slight increase from baseline to 4 weeks, with consistent results in the ITT and PP populations. These trends are represented in Figure 2A,B.

Adverse events presenting as gastrointestinal symptoms were identified in three (8.6%) patients in the control group and five (13.2%) patients in the HE-LPF group. This was thought to be related to the progression of renal disease (mainly nausea due to uremic toxins) or concomitant medications (mainly constipation due to calcium supplements). In both groups, the patients reported no gastrointestinal-associated symptoms (Table 6). Adherence to the HE-LPF ONS was 96.8% after two weeks (the first 2 weeks) and 94.9% after four weeks (the final 2 weeks). The mean (SD) intake bottles of HE-LPF were 14 (1) after two weeks, 13 (2) after four weeks, and an average of 27 (3) over the entire four-week period in the ITT population.

## 4. Discussion

We evaluated the impact of low-protein renal ONSs as either a complete or a partial meal replacement designed for the dietary management of pre-dialysis CKD patients after four weeks. The main endpoint of efficacy was the change in body weight and other parameters regarding nutritional status, including BMI, body composition, waist circumference, and hand strength; none showed any significant differences between the two groups (ITT and PP populations). In the analysis of 3-day food records, the ITT population showed a significant reduction in the energy percentage obtained from protein. Concurrently, the ITT population showed a significant increase in the energy percentage obtained from fat, including SFAs, MUFAs, and EPA, while the mineral intake remained unchanged. Meanwhile, the PP population experienced a similar result and an increase in energy intake (kcal/kg/day). Recently, a systematic review indicated that a low-protein diet might delay the progression of renal function; however, concomitantly, it may increase the risk of malnutrition, as demonstrated by the reductions seen in BMI and body weight [23]. Dietary management in CKD patients is challenging due to multiple food restrictions, low appetite frequency, or other symptoms that negatively impact nutrient intake and assimilation [5,24]. According to our findings, the HE-LPF intervention increased energy intake, decreased protein intake, and did not worsen nutritional status.

Safety endpoints did not exhibit any changes in biochemical markers. Renal function was maintained and was not significantly different between the two groups. This result may be explained by considering the impact of weight on serum creatinine levels [25]. While the two groups did not display significant differences in body weight at either baseline or after four weeks, it is noteworthy that the HE-LPF group consistently maintained a lower body weight. Although statistically significant results were not observed, this disparity may impact creatinine levels. Additionally, implementing a low-protein diet in individuals with a BMI below 18 presents specific challenges, primarily due to the heightened risk of protein–energy wasting [5]. Furthermore, there is a lack of substantial randomized controlled trials implementing low-protein diets in populations with obesity, i.e., where BMI exceeds 30 [26]. Consequently, we excluded individuals with a BMI below 18 and those with a BMI exceeding 30 from the study to reduce potential biases.

Furthermore, implementing an LPD may increase fat and carbohydrate intake, possibly affecting glycemic control or lipid profile metabolic markers. The results of metabolic markers remained stable, presumably due to either the favorable fatty acid profile or the replacement of the protein-rich foods using an HE-LPF as an interventional ONS. In our study, the analysis of the 3 day food records showed that the intake of MUFAs and EPA increased, as did that of SFAs. Evidence suggests that a MUFA-rich diet is beneficial in regulating fat oxidation, weight maintenance, and cholesterol metabolism [27]. A 13 article meta-analysis indicated that rich omega-3 fatty acids significantly decreased cardiometabolic parameters, including cholesterol and triglycerides [28]. In the 24 h urine analysis, while no statistically significant difference was observed between the two groups, we noticed a trend in the HE-LPF group toward decreased proteinuria after four weeks. Recent reports have suggested that pharmacological treatments, in synergy with dietary modifications, can enhance the care of CKD patients. For example, a protein-restricted diet has been shown to have an anti-proteinuric effect that complements ACE inhibitors or ARBs [29]. In our study, blood pressure medication remained unchanged, and there appeared to be a decreasing trend in proteinuria. This may suggest some benefit of the combined reduction in protein intake due to ONS application in the HE-LPF group, along with medication.

Adherence to the investigational product was perfect, with only a slight reduction in the last two weeks of the study compared to the first 2 week period. This result is also promising for applications in clinical practice. Uremic symptoms in CKD patients, particularly those not dialyzed, elicit taste changes and reduced food appeal [24]. This clinical reality may impact the usual food for this patient population, making ONSs a feasible nutritional management tool. In addition to high adherence to the prescribed units of the ONSs, reports of adverse effects from patients were low overall (less than 10%), mild to moderate in severity, and gastrointestinal (nausea and constipation) in nature. Gastrointestinal symptoms are a factor known to impair the provision of ONSs in CKD patients, with occurrences of nausea and constipation considered typical in uremic pre-dialysis patients [30]. Therefore, these adverse events are difficult to interpret. This is particularly pertinent since the same events occurred in both groups, suggesting that such symptoms may be related to the progression of CKD rather than the ONSs used.

There were several limitations to this study. First, the current study may have been too short in its relative duration and could not improve nutritional status. Previous studies have reported that body weight and BMI using renal-specific ONSs for six months were enhanced in patients with advanced CKD [31]. The ability to significantly increase energy intake to the extent that anthropometric indicators are also improved may take time in patients with end-stage renal disease who are at risk of PEW. Second, the study did not find a significant change between the two groups regarding body composition. However, it is worth noting that past research has suggested a potential underlying association between physical activity, especially exercise training, and nutritional supplementation, leading to increased muscle mass [32]. While we did not observe significant changes in body composition in this study, we cannot rule out the possibility of future research exploring the interplay between exercise training and nutritional supplementation, particularly in influencing muscle quality. Third, the subjects were instructed to implement a low-protein diet before being enrolled. This may have caused a risk of bias regarding the study procedures, which may explain why no significant differences were seen in the Maroni equation. Additionally, this study was conducted in a single center and may not be fully generalizable for other populations. Future research involving HE-LPF application with recruitment in multiple centers should still be conducted, including a long-term follow-up period and an exploration of the impact of physical activity.

## 5. Conclusions

Using an HE-LPF as a meal replacement in CKD patients effectively decreased the percentage of energy derived from protein while increasing the percentage of energy derived from fat. No significant differences were observed in body weight, body composition, grip strength, renal function, serum electrolytes, or metabolic markers between the two groups. The HE-LPF group showed no adverse effects and had 94.9% adherence at week 4. Thus, using a HE-LPF as a complete or partial meal replacement could be a feasible option regarding its safety regarding LPD control in pre-dialysis CKD patients.

## Figures and Tables

**Table 1:** Baseline demographic data in the intention-to-treat population.

Parameter	HE-LPF Group (*n* = 38)	Control Group (*n* = 35)	*p*-Value
Male gender, n (%)	22 (57.9)	22 (62.9)	0.665
Age (years), mean (SD)	56.4 (12.9)	53.5 (10.2)	0.230
eGFR (mL/min/1.73 m2), mean (SD)	16.0 (8.5)	17.3 (9.5)	0.585
CKD stage, n (%)			0.314
Stage 3	3 (7.9)	5 (14.3)	
Stage 4	12 (31.6)	15 (42.9)	
Stage 5	23 (60.5)	15 (42.9)	
Primary cause of CKD, n (%)			0.246
Diabetic nephropathy	11 (28.9)	13 (37.1)	
Glomerular nephropathy	9 (23.7)	2 (5.7)	
Vascular nephropathy/HTN	11 (28.9)	12 (34.3)	
Polycystic kidney disease	5 (13.2)	5 (14.3)	
Other	2 (5.3)	3 (8.6)	
Comorbidities, n (%)			
Diabetes	11 (28.9)	13 (37.1)	0.457
Hypertension	11 (28.9)	12 (34.3)	0.812
Concomitant medications, n (%)			
ACE inhibitors/ARBs	9 (23.7)	9 (25.3)	1.000
Keto-analogues	12 (31.6)	12 (34.3)	1.000
Body weight (kg), mean (SD)	62.7 (11.6)	66.5 (11.1)	0.185
Body mass index (kg/m2), mean (SD)	23.3 (3.8)	24.1 (3.2)	0.258
Grip strength (kg), mean (SD)	28.6 (10.6)	32.8 (11.4)	0.092
Vital signs			
Systolic blood pressure (mm Hg), mean (SD)	136.6 (22.1)	135.9 (16.5)	0.732
Diastolic blood pressure (mm Hg), mean (SD)	76.0 (14.9)	80 (12.8)	0.208
Heart rate (bpm), mean (SD)	75.9 (12.1)	78.6 (11.8)	0.281
Respiratory rate (bpm), mean (SD)	19.0 (5.9)	18.1 (5.5)	0.458

The data are presented as *n* (%) or the mean (SD). Statistical differences between the two groups were assessed using the Mann–Whitney U test or chi-squared test/Fisher exact test.

**Table 2:** Anthropometric indicators in the intention-to-treat population.

Parameter	HE-LPF Group (*n* = 38)	Control Group (*n* = 35)	*p*-Value
Body weight change (kg)			
Change from baseline to Week 2 ^a^	0.1 (1.4)	0.1 (1.4)	0.921
Change from baseline to Week 4 ^a^	0.0 (1.8)	0.1 (1.3)	0.685
Body weight (kg)			0.153
Baseline	62.7 (11.6)	66.5 (11.1)	
Week 2	62.8 (11.7)	66.6 (11.2)	
Week 4	62.7 (11.5)	66.6 (11.0)	
Body mass index (kg/m^2^)			0.266
Baseline	23.3 (3.8)	24.1 (3.2)	
Week 2	23.3 (3.8)	24.2 (3.3)	
Week 4	23.2 (3.8)	24.2 (3.2)	
Body fat (%)			0.157
Baseline	20.8 (6.8)	23.1 (8.1)	
Week 2	20.9 (7.5)	23.3 (8.2)	
Week 4	20.0 (8.3)	22.9 (8.0)	
Fat mass (kg)			0.087
Baseline	13.0 (5.4)	15.3 (6.5)	
Week 2	13.1 (6.1)	15.5 (6.7)	
Week 4	12.4 (6.2)	15.2 (6.5)	
Muscle mass (kg)			0.472
Baseline	46.0 (8.7)	47.5 (8.7)	
Week 2	45.9 (8.9)	47.3 (8.4)	
Week 4	46.2 (9.0)	47.6 (8.4)	
Fat free mass (kg)			0.460
Baseline	48.6 (9.0)	50.3 (9.1)	
Week 2	48.6 (9.3)	50.1 (8.7)	
Week 4	48.8 (9.4)	50.4 (8.7)	
Extracellular water (kg)			0.459
Baseline	14.0 (2.4)	14.4 (2.3)	
Week 2	13.9 (2.6)	14.3 (2.2)	
Week 4	14.0 (2.5)	14.4 (2.2)	
Waist circumference (cm)			0.269
Baseline	84.4 (10.5)	86.1 (9.3)	
Week 4	83.6 (9.9)	87.0 (9.8)	
Grip strength (kg)			0.129
Baseline	28.6 (10.6)	32.8 (11.4)	
Week 2	29.0 (10.4)	32.6 (12.3)	
Week 4	28.5 (10.4)	32.6 (11.9)	

The data are presented as the mean (SD); *p*-values were calculated using the mixed model. ^a^ *p*-values were calculated using ANCOVA.

**Table 3:** Three-day food records from baseline to weeks 2 and 4 in the ITT and PP populations.

Parameter	HE-LPF Group			Control Group			*p*-Value
	Baseline	Week 2	Week 4	Baseline	Week 2	Week 4	
ITT Population							
Energy (kcal/day)	1560 (318)	1702 (400)	1756 (339)	1671 (303)	1651 (317)	1569 (323)	0.531
Energy (kcal/kg BW/day)	25.4 (6.2)	27.6 (6.6)	28.6 (6.8)	25.6 (5.5)	25.4 (6.0)	24.1 (5.6)	0.093
Protein (g/kg BW/day)	0.78 (0.20)	0.76 (0.21)	0.80 (0.21)	0.80 (0.21)	0.82 (0.23)	0.74 (0.20)	0.898
Protein (% of energy)	12.2 (2.4)	10.9 (1.6)	11.1 (1.8)	12.4 (2.3)	12.8 (3.2)	12.3 (2.9)	0.021 *
CHO (% of energy)	51.2 (6.3)	51.2 (6.0)	51.5 (5.7)	53.2 (6.9)	52.5 (6.1)	52.9 (7.3)	0.216
Fat (% of energy)	36.6 (5.2)	38.0 (5.6)	37.3 (5.0)	34.4 (6.1)	34.7 (5.3)	34.8 (5.8)	0.010 *
SFA (g)	15.3 (4.5)	18.2 (4.6)	18.4 (4.8)	15.8 (5.2)	15.2 (4.5)	15.1 (4.4)	0.032 *
MUFA (g)	17.3 (5.9)	25.3 (6.3)	25.3 (5.6)	18.9 (7.1)	17.6 (5.6)	16.8 (4.8)	<0.001 *
PUFA (g)	25.5 (8.3)	27.2 (9.1)	27.8 (8.7)	25.3 (8.7)	25.1 (9.2)	23.0 (7.2)	0.160
EPA (mg)	68 (130)	231 (236)	134 (112)	49 (60)	70 (153)	108 (179)	0.005 *
DHA (mg)	168 (233)	313 (402)	176 (175)	109 (78)	173 (240)	246 (320)	0.307
Fiber (g)	12.2 (4.0)	12.2 (3.8)	12.4 (4.6)	13.5 (5.6)	14.0 (6.0)	12.3 (4.5)	0.264
Sodium (mg)	1526 (910)	1343 (574)	1472 (733)	1533 (568)	1525 (711)	1393 (613)	0.787
Potassium (mg)	1383 (362)	1458 (436)	1462 (545)	1596 (542)	1570 (535)	1347 (431)	0.443
Calcium (mg)	329 (147)	431 (177)	427 (142)	402 (238)	392 (225)	365 (187)	0.753
Phosphorus (mg)	592 (158)	655 (194)	696 (219)	674 (185)	674 (224)	616 (171)	0.845
Magnesium (mg)	143 (37)	173 (59)	167 (51)	173 (70)	164 (58)	146 (50)	0.999
PP Population							
Energy (kcal/day)	1600 (292)	1754 (382)	1817 (287)	1671 (303)	1651 (317)	1569 (323)	0.153
Energy (kcal/kg BW/day)	26.0 (6.2)	28.4 (6.6)	29.5 (6.6)	25.6 (5.5)	25.4 (6.0)	24.1 (5.6)	0.028 *
Protein (g/kg BW/day)	0.78 (0.20)	0.77 (0.22)	0.82 (0.20)	0.80 (0.21)	0.82 (0.23)	0.74 (0.20)	0.952
Protein (% of energy)	11.9 (2.0)	10.6 (1.4)	11.0 (1.3)	12.4 (2.3)	12.8 (3.2)	12.3 (2.9)	0.007 *
CHO (% of energy)	51.9 (5.8)	51.2 (6.0)	51.6 (4.5)	53.2 (6.9)	52.5 (6.1)	52.9 (7.3)	0.326
Fat (% of energy)	36.2 (5.0)	38.2 (5.5)	37.4 (4.3)	34.4 (6.1)	34.7 (5.3)	34.8 (5.8)	0.013 *
SFA (g)	15.4 (4.1)	18.6 (4.2)	18.9 (4.2)	15.8 (5.2)	15.2 (4.5)	15.1 (4.4)	0.008 *
MUFA (g)	17.5 (5.4)	26.0 (5.6)	26.1 (4.6)	18.9 (7.1)	17.6 (5.6)	16.8 (4.8)	<0.001 *
PUFA (g)	26.2 (8.1)	28.6 (8.2)	28.7 (8.4)	25.3 (8.7)	25.1 (9.2)	23.0 (7.2)	0.045 *
EPA (mg)	58 (118)	233 (242)	145 (116)	49 (60)	70 (153)	108 (179)	0.008 *
DHA (mg)	142 (205)	311 (412)	193 (182)	109 (78)	173 (240)	246 (320)	0.371
Fiber (g)	12.5 (4.1)	12.8 (3.7)	13.0 (4.0)	13.5 (5.6)	14.0 (6.0)	12.3 (4.5)	0.563
Sodium (mg)	1508 (885)	1376 (590)	1521 (757)	1533 (568)	1525 (711)	1393 (613)	0.914
Potassium (mg)	1404 (325)	1512 (421)	1539 (536)	1596 (542)	1570 (535)	1347 (431)	0.835
Calcium (mg)	322 (137)	446 (185)	449 (129)	402 (238)	392 (225)	365 (187)	0.536
Phosphorus (mg)	596 (156)	676 (196)	718 (217)	674 (185)	674 (224)	616 (171)	0.821
Magnesium (mg)	142 (31)	179 (60)	176 (47)	173 (70)	164 (58)	146 (50)	0.621

The data are presented as the mean (SD); *p*-values were calculated using the mixed model; * *p* < 0.05.

**Table 4:** Biochemical indicators from baseline to weeks 2 and 4 in the intention-to-treat population.

Parameter	HE-LPF Group (*n* = 38)			Control Group (*n* = 35)			*p*-Value
	Baseline	Week 2	Week 4	Baseline	Week 2	Week 4	
Renal function							
eGFR (mL/min/1.73 m^2^)	16.0 (8.5)	15.9 (8.3)	15.2 (8.2)	17.3 (9.5)	16.7 (9.1)	17.4 (9.5)	0.480
BUN (mg/dL)	58.4 (22.8)	56.6 (22.4)	58.6 (22.3)	54.8 (29.7)	57.7 (31.9)	56.9 (29.8)	0.818
Creatinine (mg/dL)	4.7 (2.2)	4.8 (2.4)	4.9 (2.4)	4.9 (3.1)	5.0 (3.1)	5.0 (3.3)	0.797
Serum electrolytes							
Sodium (mEq/L)	138.9 (3.7)	138.9 (3.7)	139.1 (3.3)	138.7 (3.5)	138.6 (3.1)	138.6 (3.1)	0.635
Chloride (mEq/L) ^a^	106.3 (4.5)	105.9 (5.0)	106.4 (4.0)	106.9 (6.7)	105.4 (4.2)	105.6 (4.3)	0.806
Potassium (mEq/L)	4.8 (0.7)	4.7 (0.7)	4.8 (0.6)	4.6 (0.5)	4.6 (0.4)	4.7 (0.5)	0.264
Calcium (mg/dL)	8.8 (0.9)	8.7 (0.7)	8.8 (0.8)	9.0 (1.0)	8.9 (0.9)	9.0 (0.9)	0.213
Phosphorus (mg/dL)	4.3 (0.7)	4.3 (1.0)	4.3 (0.7)	4.2 (1.1)	4.3 (1.3)	4.3 (1.2)	0.774
Magnesium (mg/dL)	2.2 (0.4)	—	2.3 (0.3)	2.2 (0.3)	—	2.2 (0.4)	0.902
Plasma protein							
Albumin (g/dL)	4.2 (0.3)	4.1 (0.3)	4.2 (0.3)	4.2 (0.3)	4.1 (0.3)	4.1 (0.3)	0.780
Prealbumin (g/L)	0.3 (0.1)	0.3 (0.1)	0.3 (0.1)	0.3 (0.1)	0.3 (0.0)	0.3 (0.1)	0.554
Total protein (g/dL)	7.0 (0.6)	6.9 (0.6)	7.0 (0.5)	6.9 (0.6)	6.8 (0.6)	6.8 (0.5)	0.337
CRP (mg/dL)	0.2 (0.2)	—	0.2 (0.3)	0.1 (0.2)	—	0.3 (0.7)	0.752
Metabolic markers							
Cholesterol (mg/dL)	163.6 (38.1)	—	162.3 (37.5)	165.2 (41.3)	—	162.8 (40.7)	0.907
Triglyceride (mg/dL)	109.8 (52.5)	—	126.2 (95.9)	134.5 (55.9)	—	125.6 (51.8)	0.401
LDL-C (mg/dL)	90.3 (30.7)	—	86.3 (25.5)	94.9 (32.6)	—	93.3 (32.5)	0.412
Uric acid (mg/dL)	6.2 (1.9)	—	6.0 (1.8)	6.2 (1.9)	—	5.8 (1.8)	0.778
HbA1c (%)	5.8 (0.6)	—	5.8 (0.6)	6.1 (1.3)	—	6.0 (1.2)	0.208
24 h urine collection							
24 h urine protein (g)	1.4 (1.6)	1.5 (1.7)	1.3 (1.3)	1.7 (2.2)	1.6 (1.7)	1.9 (2.4)	0.411
24 h UUN (g)	6.7 (2.8)	6.7 (2.3)	6.5 (2.3)	7.1 (3.9)	6.7 (2.6)	7.4 (3.4)	0.434
Dietary protein intake (g/day)	55.5 (18.5)	55.7 (15.6)	54.0 (15.6)	59.2 (25.5)	56.5 (16.9)	61.2 (22.5)	0.314
Dietary protein intake (g/kg BW/day)	0.89 (0.26)	0.89 (0.20)	0.86 (0.22)	0.89 (0.38)	0.86 (0.25)	0.93 (0.32)	0.835

The data are presented as the mean (SD); *p*-values were calculated using the mixed model. ^a^ HE-LPF Group (*n =* 37) and Control Group (*n* = 34), respectively.

**Table 5:** Biochemical indicators from baseline to weeks 2 and 4 in the per-protocol population.

Parameter	HE-LPF Group (*n* = 33)			Control Group (*n* = 35)			*p*-Value
	Baseline	Week 2	Week 4	Baseline	Week 2	Week 4	
Renal function							
eGFR (mL/min/1.73 m^2^)	16.4 (8.8)	16.3 (8.6)	15.8 (8.5)	17.3 (9.5)	16.7 (9.1)	17.4 (9.5)	0.656
BUN (mg/dL)	57.2 (21.4)	54.0 (20.8)	56.8 (21.1)	54.8 (29.7)	57.7 (31.9)	56.9 (29.8)	0.944
Creatinine (mg/dL)	4.6 (2.3)	4.7 (2.5)	4.8 (2.5)	4.9 (3.1)	5.0 (3.1)	5.0 (3.3)	0.728
Serum electrolytes							
Sodium (mEq/L)	139.5 (2.9)	139.6 (3.1)	139.6 (3.0)	138.7 (3.5)	138.6 (3.1)	138.6 (3.1)	0.188
Chloride (mEq/L) ^a^	106.7 (3.9)	106.3 (4.5)	106.8 (3.9)	106.9 (6.7)	105.4 (4.2)	105.6 (4.3)	0.510
Potassium (mEq/L)	4.7 (0.7)	4.7 (0.7)	4.8 (0.6)	4.6 (0.5)	4.6 (0.4)	4.7 (0.5)	0.543
Calcium (mg/dL)	8.8 (0.9)	8.7 (0.7)	8.8 (0.8)	9.0 (1.0)	8.9 (0.9)	9.0 (0.9)	0.255
Phosphorus (mg/dL)	4.3 (0.7)	4.3 (1.0)	4.3 (0.7)	4.2 (1.1)	4.3 (1.3)	4.3 (1.2)	0.906
Magnesium (mg/dL)	2.3 (0.4)		2.3 (0.4)	2.2 (0.3)		2.2 (0.4)	0.595
Plasma protein							
Albumin (g/dL)	4.2 (0.3)	4.1 (0.3)	4.2 (0.3)	4.2 (0.3)	4.1 (0.3)	4.1 (0.3)	0.826
Prealbumin (g/L)	0.3 (0.1)	0.3 (0.1)	0.3 (0.1)	0.3 (0.1)	0.3 (0.0)	0.3 (0.1)	0.280
Total protein (g/dL)	7.0 (0.6)	6.9 (0.6)	7.0 (0.6)	6.9 (0.6)	6.8 (0.6)	6.8 (0.5)	0.333
CRP (mg/dL)	0.2 (0.2)		0.2 (0.3)	0.1 (0.2)		0.3 (0.7)	0.907
Metabolic markers							
Cholesterol (mg/dL)	161.5 (37.0)	—	159.5 (32.4)	165.2 (41.3)	—	162.8 (40.7)	0.702
Triglyceride (mg/dL)	108.6 (55.2)	—	112.9 (70.8)	134.5 (55.9)	—	125.6 (51.8)	0.158
LDL-C (mg/dL)	89.2 (30.0)	—	86.4 (24.7)	94.9 (32.6)	—	93.3 (32.5)	0.384
Uric acid (mg/dL)	5.9 (1.6)	—	5.8 (1.8)	6.2 (1.9)	—	5.8 (1.8)	0.775
HbA1c (%)	5.8 (0.6)	—	5.8 (0.6)	6.1 (1.3)	—	6.0 (1.2)	0.214
24 h urine collection							
24 h urine protein (g)	1.4 (1.7)	1.5 (1.7)	1.3 (1.4)	1.7 (2.2)	1.6 (1.7)	1.9 (2.4)	0.444
24 h UUN (g)	6.9 (2.7)	6.9 (2.3)	6.7 (2.3)	7.1 (3.9)	6.7 (2.6)	7.4 (3.4)	0.656
Dietary protein intake (g/day)	56.7 (17.9)	56.9 (15.5)	55.3 (15.4)	59.2 (25.5)	56.5 (16.9)	61.2 (22.5)	0.510
Dietary protein intake (g/kg BW/day)	0.91 (0.25)	0.91 (0.20)	0.88 (0.22)	0.89 (0.38)	0.86 (0.25)	0.93 (0.32)	0.910

The data are presented as the mean (SD); *p*-values were calculated using the mixed model. ^a^ HE-LPF Group (*n =* 32) and Control Group (*n* = 34), respectively.

**Table 6:** Summary of gastrointestinal symptoms from baseline to weeks 2 and 4.

Parameters	HE-LPF Group (*n* = 38)	Control Group (*n* = 35)	Overall (*n* = 73)
Diarrhea	0 (0.0)	0 (0.0)	0 (0.0)
Abdominal distension	0 (0.0)	0 (0.0)	0 (0.0)
Constipation	3 (7.9)	2 (5.7)	5 (6.8)
Nausea	2 (5.3)	1 (2.9)	3 (4.1)
Vomiting	0 (0.0)	0 (0.0)	0 (0.0)

The data are presented as *n* (%).

## Data Availability

Data are unavailable due to privacy or ethical restrictions.

## Supplementary Materials

The following supporting information can be downloaded at https://www.mdpi.com/article/10.3390/nu15214506/s1, Table S1. The primary nutritional composition of the high-energy, low-protein formula; Table S2. Anthropometric indicators in the per-protocol population.

## References

[1] Kovesdy C.P. (2022). Epidemiology of chronic kidney disease: An update 2022. Kidney Int. Suppl..

[2] Chen T.K., Knicely D.H., Grams M.E. (2019). Chronic Kidney Disease Diagnosis and Management: A Review. JAMA.

[3] Kalantar-Zadeh K., Jafar T.H., Nitsch D., Neuen B.L., Perkovic V. (2021). Chronic kidney disease. Lancet.

[4] Kalantar-Zadeh K., Fouque D. (2017). Nutritional Management of Chronic Kidney Disease. N. Engl. J. Med..

[5] Ikizler T.A., Burrowes J.D., Byham-Gray L.D., Campbell K.L., Carrero J.J., Chan W., Fouque D., Friedman A.N., Ghaddar S., Goldstein-Fuchs D.J. (2020). KDOQI Clinical Practice Guideline for Nutrition in CKD: 2020 Update. Am. J. Kidney Dis..

[6] Kistler B.M., Moore L.W., Benner D., Biruete A., Boaz M., Brunori G., Chen J., Drechsler C., Guebre-Egziabher F., Hensley M.K. (2021). The International Society of Renal Nutrition and Metabolism Commentary on the National Kidney Foundation and Academy of Nutrition and Dietetics KDOQI Clinical Practice Guideline for Nutrition in Chronic Kidney Disease. J. Ren. Nutr..

[7] Paes-Barreto J.G., Silva M.I., Qureshi A.R., Bregman R., Cervante V.F., Carrero J.J., Avesani C.M. (2013). Can renal nutrition education improve adherence to a low-protein diet in patients with stages 3 to 5 chronic kidney disease?. J. Ren. Nutr..

[8] Cupisti A., Gallieni M., Avesani C.M., D’Alessandro C., Carrero J.J., Piccoli G.B. (2020). Medical Nutritional Therapy for Patients with Chronic Kidney Disease not on Dialysis: The Low Protein Diet as a Medication. J. Clin. Med..

[9] Pisani A., Riccio E., Bellizzi V., Caputo D.L., Mozzillo G., Amato M., Andreucci M., Cianciaruso B., Sabbatini M. (2016). 6-tips diet: A simplified dietary approach in patients with chronic renal disease. A clinical randomized trial. Clin. Exp. Nephrol..

[10] Carrero J.J., Stenvinkel P., Cuppari L., Ikizler T.A., Kalantar-Zadeh K., Kaysen G., Mitch W.E., Price S.R., Wanner C., Wang A.Y. (2013). Etiology of the protein-energy wasting syndrome in chronic kidney disease: A consensus statement from the International Society of Renal Nutrition and Metabolism (ISRNM). J. Ren. Nutr..

[11] Schrauben S.J., Apple B.J., Chang A.R. (2022). Modifiable Lifestyle Behaviors and CKD Progression: A Narrative Review. Kidney360.

[12] MacLaughlin H.L., Friedman A.N., Ikizler T.A. (2022). Nutrition in Kidney Disease: Core Curriculum 2022. Am. J. Kidney Dis..

[13] Wu H.L., Sung J.M., Kao M.D., Wang M.C., Tseng C.C., Chen S.T. (2013). Nonprotein calorie supplement improves adherence to low-protein diet and exerts beneficial responses on renal function in chronic kidney disease. J. Ren. Nutr..

[14] Kanazawa Y., Morita S., Sonoki H., Nakao T. (2016). Effects of a novel nutritional formula specially developed for chronic kidney disease patients on protein-restricted diets: A randomized controlled trial. Ren. Replace. Ther..

[15] Montes-Delgado R., Guerrero Riscos M.A., García-Luna P.P., Martín Herrera C., Pereira Cunill J.L., Garrido Vázquez M., López Muñoz I., Suárez García M.J., Martín-Espejo J.L., Soler Junco M.L. (1998). Treatment with low-protein diet and caloric supplements in patients with chronic kidney failure in predialysis. Comparative study. Rev. Clin. Esp..

[16] Satirapoj B., Prapakorn J., Punpanich D., Pongsuparbchon C., Supasyndh O. (2016). The effect of ONCE Renal on minerals and electrolytes in predialysis patients with chronic kidney disease. Int. J. Nephrol. Renov. Dis..

[17] Mustad V.A., Hegazi R.A., Hustead D.S., Budiman E.S., Rueda R., Maki K., Powers M., Mechanick J.I., Bergenstal R.M., Hamdy O. (2020). Use of a diabetes-specific nutritional shake to replace a daily breakfast and afternoon snack improves glycemic responses assessed by continuous glucose monitoring in people with type 2 diabetes: A randomized clinical pilot study. BMJ Open Diab. Res. Care.

[18] Bowen J., Brindal E., James-Martin G., Noakes M. (2018). Randomized Trial of a High Protein, Partial Meal Replacement Program with or without Alternate Day Fasting: Similar Effects on Weight Loss, Retention Status, Nutritional, Metabolic, and Behavioral Outcomes. Nutrients.

[19] Gulati S., Misra A., Tiwari R., Sharma M., Pandey R.M., Yadav C.P. (2017). Effect of high-protein meal replacement on weight and cardiometabolic profile in overweight/obese Asian Indians in North India. Br. J. Nutr..

[20] Levey A.S., Stevens L.A., Schmid C.H., Zhang Y.L., Castro A.F., Feldman H.I., Kusek J.W., Eggers P., Van Lente F., Greene T. (2009). A new equation to estimate glomerular filtration rate. Ann. Intern. Med..

[21] Maroni B.J., Steinman T.I., Mitch W.E. (1985). A method for estimating nitrogen intake of patients with chronic renal failure. Kidney Int..

[22] Faul F., Erdfelder E., Lang A.-G., Buchner A. (2007). G*Power 3: A flexible statistical power analysis program for the social, behavioral, and biomedical sciences. Behav. Res. Methods.

[23] Yue H., Zhou P., Xu Z., Liu L., Zong A., Qiu B., Liu W., Jia M., Du F., Xu T. (2020). Effect of low-protein diet on kidney function and nutrition in nephropathy: A systematic review and meta-analysis of randomized controlled trials. Clin. Nutr..

[24] Carrero J.J. (2011). Mechanisms of altered regulation of food intake in chronic kidney disease. J. Ren. Nutr..

[25] Bjornsson T.D. (1979). Use of Serum Creatinine Concentrations to Determine Renal Function1. Clin. Pharmacokinet..

[26] Torreggiani M., Wang A.Y., Fois A., Piccoli G.B. (2023). Personalized Low-Protein Diet Prescription in CKD Population: Merging Evidence From Randomized Trials With Observational Data. Semin. Nephrol..

[27] Hammad S., Pu S., Jones P.J. (2016). Current Evidence Supporting the Link Between Dietary Fatty Acids and Cardiovascular Disease. Lipids.

[28] Fazelian S., Moradi F., Agah S., Hoseini A., Heydari H., Morvaridzadeh M., Omidi A., Pizarro A.B., Ghafouri A., Heshmati J. (2021). Effect of omega-3 fatty acids supplementation on cardio-metabolic and oxidative stress parameters in patients with chronic kidney disease: A systematic review and meta-analysis. BMC Nephrol..

[29] Giannese D., D’Alessandro C., Panichi V., Pellegrino N., Cupisti A. (2023). Nutritional Treatment as a Synergic Intervention to Pharmacological Therapy in CKD Patients. Nutrients.

[30] Almeras C., Argilés A. (2009). The general picture of uremia. Semin. Dial..

[31] Kelly O.J., Huang M.C., Liao H.Y., Lin C.C., Tung T.Y., Cheng R.W., Wang M.Y., Yalawar M., Hwang S.J. (2021). A Low-Protein Diet with a Renal-Specific Oral Nutrition Supplement Helps Maintain Nutritional Status in Patients with Advanced Chronic Kidney Disease. J. Pers. Med..

[32] Ekramzadeh M., Santoro D., Kopple J.D. (2022). The Effect of Nutrition and Exercise on Body Composition, Exercise Capacity, and Physical Functioning in Advanced CKD Patients. Nutrients.

