# Supplementary material for: Efficacy and Safety of a High-Energy, Low-Protein Formula Replacement Meal for Pre-Dialysis Chronic Kidney Disease Patients: A Randomized Controlled Trial
Source: Nutrients. 2023 Oct 24;15(21):4506. doi: 10.3390/nu15214506 (PMC10648072; doi:10.3390/nu15214506)
Supplement: Supplementary file 1 [file nutrients-15-04506-s001.zip › nutrients-2644201-supplementary.pdf]

Table S1. The primary nutritional composition of the high-energy, low-protein formula.

**Contents**	**per200 mL/Bot**
Energy (kcal)	400
Protein(g)	6
Carbohydrates (g)	55.2
Fat (g)	17.8
Dietary fiber (g)	2.4
Saturated Fatty Acids (g)	5.4
Polyunsaturated Fatty Acids (g)	2.9
Monounsaturated Fatty Acids (g)	9.4
Eicosapentaenoic Acid (mg)	84
Docosahexaenoic Acid (mg)	38
Sodium (mg)	136
Potassium (mg)	200
Calcium (mg)	168
Phosphorus (mg)	110
Magnesium (mg)	40

Table S2. Anthropometric indicators in the per-protocol population.

Parameter	HE-LPF Group (*n* = 33)	Control Group (*n* = 35)	*p*-Value
Body weight change (kg)			
Change from baseline to Week 2 [a]	0.1 (1.5)	0.1 (1.4)	0.974
Change from baseline to Week 4 [a]	0.2 (1.8)	0.1 (1.3)	0.885
Body weight (kg)			0.234
Baseline	63.1 (12.0)	66.5 (11.1)	
Week 2	63.2 (12.2)	66.6 (11.2)	
Week 4	63.3 (11.9)	66.6 (11.0)	
Body Mass Index (kg/m^2)			0.494
Baseline	23.6 (3.9)	24.1 (3.2)	
Week 2	23.6 (3.9)	24.2 (3.3)	
Week 4	23.6 (3.8)	24.2 (3.2)	
Body fat (%)			0.373
Baseline	21.5 (6.8)	23.1 (8.1)	
Week 2	21.8 (7.3)	23.3 (8.2)	
Week 4	20.9 (8.3)	22.9 (8.0)	
Fat mass (kg)			0.218
Baseline	13.6 (5.5)	15.3 (6.5)	
Week 2	13.8 (6.1)	15.5 (6.7)	
Week 4	13.1 (6.3)	15.2 (6.5)	
Muscle mass (kg)			0.431
Baseline	45.9 (8.9)	47.5 (8.7)	
Week 2	45.6 (9.3)	47.3 (8.4)	
Week 4	45.9 (9.4)	47.6 (8.4)	
Fat free mass (kg)			0.421
Baseline	48.5 (9.3)	50.3 (9.1)	
Week 2	48.2 (9.8)	50.1 (8.7)	
Week 4	48.5 (9.8)	50.4 (8.7)	
Extracellular water (kg)			0.596
Baseline	14.2 (2.5)	14.4 (2.3)	
Week 2	14.0 (2.7)	14.3 (2.2)	
Week 4	14.0 (2.7)	14.4 (2.2)	
Waist circumference (cm)			0.484
Baseline	85.2 (10.8)	86.1 (9.3)	
Week 4	84.4 (10.2)	87.0 (9.8)	
Grip Strength (kg)			0.075
Baseline	27.8 (10.2)	32.8 (11.4)	
Week 2	28.3 (10.3)	32.6 (12.3)	
Week 4	27.5 (10.2)	32.6 (11.9)	

The data are presented as the mean (SD); *p*-values were calculated using the mixed model. [a] *p*-values were calculated with ANCOVA.
